# Robust Silica-Bacterial Cellulose Composite Aerogel Fibers for Thermal Insulation Textile

**DOI:** 10.3390/gels7030145

**Published:** 2021-09-17

**Authors:** Huazheng Sai, Meijuan Wang, Changqing Miao, Qiqi Song, Yutong Wang, Rui Fu, Yaxiong Wang, Litong Ma, Yan Hao

**Affiliations:** 1 School of Chemistry and Chemical Engineering, Inner Mongolia University of Science & Technology, Baotou 014010, China; wmjbest1014@163.com (M.W.); qingmc@163.com (C.M.); songqiqiaa@163.com (Q.S.); wangyut@163.com (Y.W.); wangyaxiong2021@126.com (Y.W.); mlt0916@126.com (L.M.); haoyannk@163.com (Y.H.); 2 Inner Mongolia Engineering Research Center of Comprehensive Utilization of Bio-Coal Chemical Industry, Inner Mongolia University of Science & Technology, Baotou 014010, China; 3 Inner Mongolia Key Laboratory of Coal Chemical Engineering & Comprehensive Utilization, Inner Mongolia University of Science & Technology, Baotou 014010, China

**Keywords:** fibrous aerogel, nanoscale interpenetrating network, secondary shaping, strength, thermal properties

## Abstract

Aerogels are nanoporous materials with excellent properties, especially super thermal insulation. However, owing to their serious high brittleness, the macroscopic forms of aerogels are not sufficiently rich for the application in some fields, such as thermal insulation clothing fabric. Recently, freeze spinning and wet spinning have been attempted for the synthesis of aerogel fibers. In this study, robust fibrous silica-bacterial cellulose (BC) composite aerogels with high performance were synthesized in a novel way. Silica sol was diffused into a fiber-like matrix, which was obtained by cutting the BC hydrogel and followed by secondary shaping to form a composite wet gel fiber with a nanoscale interpenetrating network structure. The tensile strength of the resulting aerogel fibers reached up to 5.4 MPa because the quantity of BC nanofibers in the unit volume of the matrix was improved significantly by the secondary shaping process. In addition, the composite aerogel fibers had a high specific area (up to 606.9 m^2^/g), low density (less than 0.164 g/cm^3^), and outstanding hydrophobicity. Most notably, they exhibited excellent thermal insulation performance in high-temperature (210 °C) or low-temperature (−72 °C) environments. Moreover, the thermal stability of CAFs (decomposition temperature was about 330 °C) was higher than that of natural polymer fiber. A novel method was proposed herein to prepare aerogel fibers with excellent performance to meet the requirements of wearable applications.

## 1. Introduction

Aerogels are materials with excellent features, such as large specific surface area (500–1200 m^2^/g), high porosity (80–99.8%), and low density (0.003–0.5 g/cm^3^) [1], which make them readily applicable in adsorption [2,3], heat preservation [4,5], and catalysis [6,7]. However, as ultra-porous materials, aerogels are often highly brittle, especially in the case of a three-dimensional gel skeleton composed of nanoparticles, e.g., silica and other inorganic oxide aerogels [8]. This poor mechanical property is mainly due to the very small connection area between the nanoparticles that make up the gel skeleton [9]. Hence, the utilization and promotion of aerogels are severely restricted by their low mechanical strength.

Researchers worldwide have mainly focused on improving the mechanical properties of aerogel materials through precursor regulation and external doping. Methods of regulating gel precursors include increasing the quantity of precursors [10], using precursors containing inert groups [11,12,13], using precursors with flexible chains [14,15,16], and using precursors containing polymer monomers to construct bimolecular chain cross-linking network structures [17,18] to reduce the content of rigid –Si–O–Si–, giving the gel skeleton excellent flexibility to resist the impact of external forces. The external doping strategy includes a mixed extrusion of aerogel powder and long fiber [19], dispersion fiber doping [20], reinforcement by fiber felt [21,22] and isometric growth of polymers on the aerogel skeleton [23,24] to connect different areas of the skeleton or expend the connection area of the nanoparticles. These two strategies improve the mechanical properties to a great extent, but they are mainly applied to aerogel blocks, sheets, and blankets.

At present, the excellent thermal insulation performance of aerogel materials makes them suitable for application in thermal insulation clothing and other fabrics, which would require the processing and manufacture of aerogels as high-strength fibers. Aerogel fibers not only enrich the morphology of aerogels but also expand the applicability of aerogels in different fields and contexts. In addition to thermal insulation applications [25,26], aerogel fibers could also be used in other fields, such as adsorption [27,28,29], biological sensing [30], and supercapacitors [31]. As a result of these potential advantages, it is essential to develop different strategies to obtain high-strength aerogel fibers.

Freeze spinning and wet spinning are the most commonly reported methods used to prepare aerogel fibers [32]. The raw materials of the aerogel fibers are typically synthetic polymers [33], natural polymers [34], graphene oxide [35], or their composites [36]. Although some studies have reported on inorganic oxide (e.g., SiO_2_) aerogels, their tensile strength generally does not exceed 0.5 MPa [27,37], which is much lower than that of the previously mentioned fibrous aerogels. This is because there are significant differences between their microstructures. As a result of the extremely limited particle connection area, the “pearl-necklace” network formed by inorganic oxide nanoparticles is weaker than the gel skeleton composite of nanofiber-like or nanosheet-like nanostructure units connected to each other [38]. Unlike direct freeze spinning or wet spinning, a hollow polymer fiber is prepared by coaxial wet spinning, and then silk fibroin, graphene oxide, etc. are poured into it. Subsequently, the fiber being freeze dried to construct a gel skeleton was achieved recently [26,36]. The tensile strength of the obtained sheath–core coaxial aerogel fibers is significantly improved compared with that of fibrous aerogels without an outer polymer layer. This preparation process, which uses coaxial wet spinning to construct the hollow polymer fiber as a protective layer, is complicated and time consuming. Therefore, it is critical to continuously explore new strategies to prepare high-strength aerogel fibers, especially inorganic oxide-based aerogel fibers with excellent thermal insulation properties.

**Figure 1:**
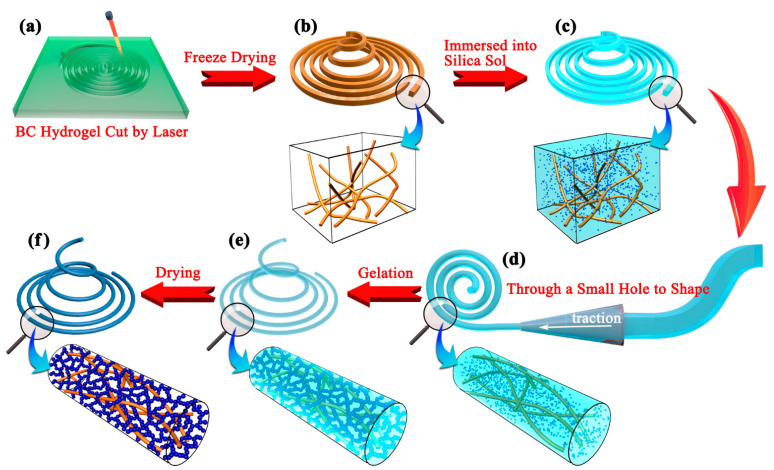
Schematic of the preparation process of CAFs. A *nata-de-coco* slice was cut by a laser (**a**), followed by freeze drying to obtain a fiber-like matrix consisting of BC nanofibers (**b**). The matrix containing silica sols (**c**) was reshaped by a small hole mold (**d**). After the silica gel skeleton was formed in the matrix (**e**), the fiber-like composite wet gel was dried at ambient pressure after hydrophobization to obtain CAFs (**f**).

In this paper, a novel method that does not use spinning is proposed for fabricating aerogel fibers with a nanoscale interpenetrating network (as shown in Figure 1), which is a structure that has been proven to significantly improve the mechanical properties of aerogel blocks or films. Bacterial cellulose (BC) hydrogel was processed into a long fiber as the matrix, and then, the silica sol was diffused into the matrix. After secondary shaping to regulate the matrix morphology at both macroscopic and microscopic levels, the nanoscale interpenetrating network structure was obtained by an in situ sol-gel reaction. Finally, silica-BC composite aerogel fibers (CAFs) were obtained by hydrophobic modification and atmospheric pressure drying. The secondary shaping process, which increases the content of BC nanofibers per unit volume of the matrix, combined with the excellent mechanical properties of BC [39], significantly improves the tensile strength of the silica aerogel fibers.

## 2. Results and Discussion

### 2.1. Synthesis of CAFs and Their Macroscopic Characteristics

**Figure 2:**
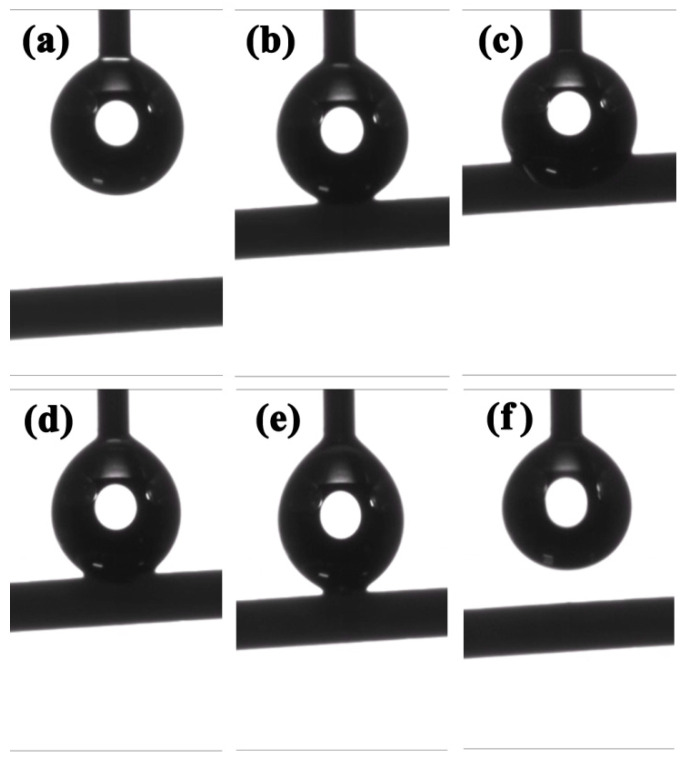
Wettability of CAF-3. (**a**–**c**) The gradual contact process between water droplets and the CAF. (**d**–**f**) The process of water droplets leaving the CAF.

The detailed preparation method was described in the section “Materials and Methods part (Section 4). The diameter of the obtained CAFs was approximately 0.7 mm, as obtained from scanning electron microscopy (SEM) images; this diameter was close to the inner diameter of the mold. This means that the sample was able to almost completely spring back in the process of atmospheric drying owing to the effective hydrophobic modification. The hydroxyl groups (including –C–OH and –Si–OH) on the surface of BC nanofibers and silica gel skeleton were replaced with the inert and hydrophobic methyl groups through the hydrophobic modification process, which inhibited the formation of new –Si–O–Si– on the surface of the gel skeleton, allowing it to spring back during the drying process. Moreover, the hydrophobic modification also endowed the CAFs with excellent hydrophobicity. As shown in Figure 2 and Video S1, the water droplet maintained its spherical shape even when the fiber was pushed against the droplet. The excellent hydrophobicity could prevent the nanoporous structure of the CAFs from being destroyed by water vapor or water droplets.

### 2.2. Microstructures

**Figure 3:**
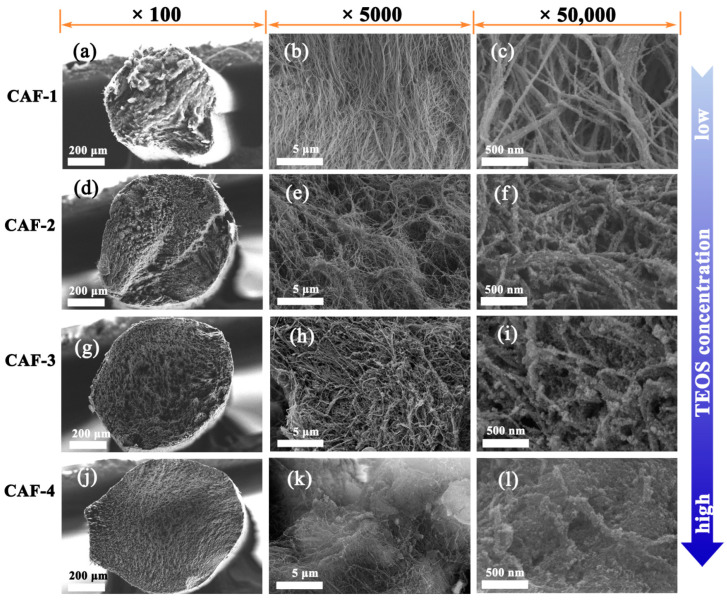
The SEM images of CAF-1 (**a**–**c**), CAF-2 (**d**–**f**), CAF-3 (**g**–**i**), and CAF-4 (**j–l**) with different magnifications.

As shown in the SEM images (Figure 3), when the precursor concentration was low, the CAF-1 was mainly composed of BC nanofiber aggregates, with only a small amount of silica nanoparticles attached to the BC by hydrogen bonding. At larger precursor concentrations, as in CAF-2 and CAF-3, the silica gel skeleton was gradually formed in the BC nanofiber network. Thus, the nanoscale interpenetrating network structure was constructed. When the precursor concentration was improved further, the silica gel skeleton became more compact, as shown in CAF-4. It can be seen from the cross-section that the diameter of the sample is approximately 0.7 mm, which is close to the inner diameter of the secondary shaping mold, indicating that the sample did not significant shrink during the atmospheric pressure drying process. The diameter of CAF-1 was slightly smaller than those of the other samples because of the lack of a rigid silica gel skeleton, which caused the sample to shrink by a certain extent during the drying process. As the gel skeleton first shrinks and then springs back during the drying process, the gel skeleton must reach a certain strength to ensure that it can effectively spring back. As a result, a certain concentration of precursors (TEOS) to construct a gel skeleton with sufficient strength is very important for the preparation of composite aerogel fibers.

In addition, the spatial distribution of BC nanofibers in CAFs was obviously denser than that of BC nanofibers without secondary shaping (Figure S2), which is beneficial for further improving the strength of the composite aerogels. Compared with our previous study, the higher density of the obtained BC nanofibers enables the samples to better retain their morphology without shrinkage, even at low concentrations of silica precursors [40]. This could be because denser BC nanofibers could more effectively resist the shrinkage caused by capillary forces during the ambient pressure drying process.

**Figure 4:**
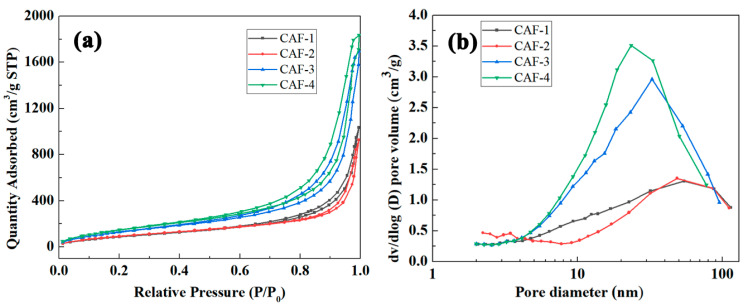
Nitrogen adsorption–desorption isotherms (**a**) and pore size distribution (**b**) of CAFs.

The nitrogen adsorption–desorption isotherms of the prepared CAFs (Figure 4a) were type IV isotherms with hysteresis loops, confirming the formation of mesoporous structures. The hysteresis loops of CAF-3 and CAF-4 were more obvious than those of CAF-1 and CAF-2. Moreover, the pore size distribution (Figure 4b) showed that CAF-3 and CAF-4 had more significant mesoporous structure characteristics than CAF-1 and CAF-2. This suggests that sufficient silica precursors are required to form a complete gel skeleton. When the concentration of silica precursor was relatively low, there was a lack of sufficient silica nanoparticles among the nanofiber network to form a gel skeleton due to the attachment of silica nanoparticles to BC nanofibers. In particular, the number of BC nanofibers per unit volume was enhanced by secondary shaping, which further enhanced the adhesion of silica particles to the BC nanofibers. Based on the results of a BET test, the specific surface areas of the samples grew from 367.9 to 606.9 m^2^/g (Table 1) with an increase in TEOS concentration, which is higher than that of PAO@ANF symbiotic aerogel fiber [28] and Kevlar aerogel fibers [25]. This is because a gel skeleton composed of inorganic oxide nanoparticles has a rougher surface compared to a gel skeleton composed of nanofibers or nanosheets.

### 2.3. Mechanical Properties

**Figure 5:**
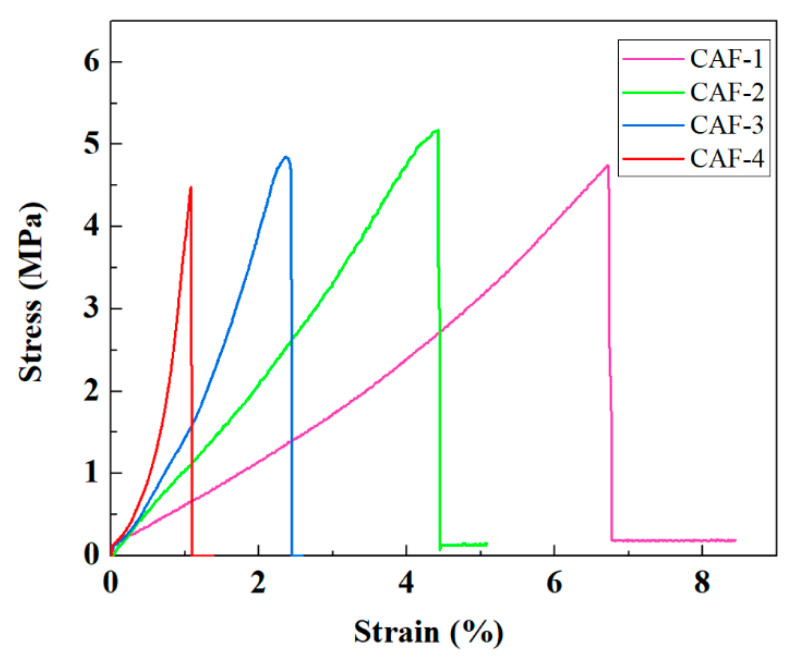
Stress–strain curves of CAFs.

High mechanical performance is crucial for a wearable thermal insulation material. The mechanical performances of CAFs prepared with different concentrations of TEOS precursor are presented in Figure 5. The stress–strain curves of CAFs obtained from tensile tests showed that the breaking stress was in the range of 4.5–5.4 MPa. All the samples showed good tensile strength, which was much higher than that of native silica aerogel fibers, and it was comparable or even superior to those of CA/PAA-SF aerogel fibers [41] (3 MPa), SF/GO aerogel fibers [36] (3.2 MPa), and PAO@ANF aerogel fibers [28] (4.56 MPa). Moreover, the curves also showed that the elongation at break of the sample decreased from 6.8% to 1.1% with increasing precursor concentration. This could be because when more and denser gel skeletons formed between the BC nanofibers, the free movement of the nanofibers was restricted, resulting in the free deformation space of the nanofiber network being compressed. At the same time, the sample density increased slightly with increasing precursor concentration because of the enhancement of silica that forms the gel skeleton in the obtained CAFs, but it was generally in a low range (Table 1).

### 2.4. Thermal Insulation

**Figure 6:**
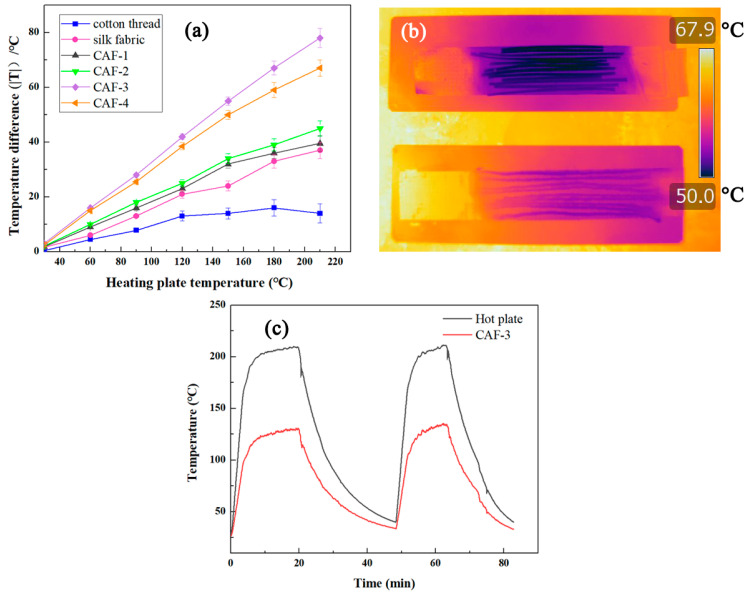
Thermal insulation properties of CAFs, silk fabric, and cotton threads. Temperature difference between the fiber surface and hot plate versus the hot plate for the single-layer mat of CAFs, silk fabric, and cotton threads (**a**). Infrared photo of one-layer mats of CAF-3 and cotton threads at high temperatures (**b**). Temperature–time curves of CAF-3 and hot plate (**c**).

The insulation performance of CAFs obtained from different concentrations of TEOS was tested under hot and cold conditions. Cotton threads and silk fabric, with similar diameters or thicknesses as those of the CAFs, were also tested under the same conditions. First, several CAFs were packed tightly and aligned in one direction to form a single-layer mat with a thickness of approximately 0.7 mm and placed on a hot plate. The thermocouple was connected to the fiber surface and the hot plate, and the temperature of the surface of CAFs (*T_f_*) was recorded during the heating of the hot plate from 30 to 200 °C. The absolute value of the temperature difference (|Δ*T*|) of the CAF surface (*T_f_*) and the hot plate were plotted against the hot plate temperature (*T_h_*). As shown in Figure 6a, while heating the hot plate from 30 to 200 °C, the temperature difference of the one-layer CAF mat was always higher than those of the one-layer silk fabric mat and cotton fabric mat. When *T_h_* was 120 °C, the CAF mat temperature reached 79 °C, and the temperatures of the silk and cotton fabric mats reached 101.3 °C and 107 °C, respectively. The higher the temperature difference (|Δ*T*|), the better the thermal insulation of the studied materials. Hence, the thermal insulation properties of CAFs are superior to those of silk fabrics and cotton threads. Meanwhile, for visually comparing the thermal insulation difference between the CAF fabric and cotton threads at the same heat source temperature (80 °C), a layer of CAF-3 formed by approximately 10 samples and a layer of 10 cotton threads of the same length were heated at the same time. After the temperature was stable, an infrared camera was used to capture photographs (Figure 6b), which showed that the temperature of the cotton fabric was higher than that of the CAF fabric, intuitively proving that the CAFs have excellent thermal insulation properties.

As seen in Figure 6a, with an increase in the amounts of TEOS in CAF-1, CAF-2, and CAF-3, the thermal insulation performances of the corresponding CAFs were gradually enhanced, owing to the complete gel skeleton gradually forming with increasing solid concentration in the aerogel. However, when the concentration of the silica precursor increased further (CAF-4), the thermal insulation performance of CAF-4 decreased because of the high content of the solid phase and because heat is more easily transmitted within the solid phase. At the same time, when the temperature was high, the slope of |Δ*T*| to *T_h_* decreased slightly with an increase in temperature, implying that the proportion of thermal radiation on heat transfer rose gradually at high temperatures. In fact, the thermal insulation mechanism of aerogels mainly depended on the blocking of thermal convection and heat conduction, not thermal radiation. Figure 6c shows the precise values of temperature change on the surface of the hot plate (*T_h_*) and aerogel fibers (CAF-3) during the heating process of the hot plate. The surface temperature of CAF-3 varied from 28 to 131 °C as the temperature of the hot plate rose from 30 to 210 °C. When *T_h_* was stable at 210 °C, the |Δ*T*| of the hot plate and CAF-3 was approximately 80 °C. When CAF-3 was heated again after a heating-cooling process, the |Δ*T*| showed no obvious change, indicating that the thermal insulation performance of CAF-3 was stable. The temperature–time curves for the other samples are shown in Figure S3 (in the Supporting Information) and indicate the stability of the thermal insulation.

To test the thermal insulation performance of the sample at lower temperatures, the three single-layer fiber mats of CAF-3, silk fabric, and cotton threads were placed on a sheet of iron with 2.5 cm of dry ice underneath. The temperatures of the fiber surface and iron sheet were monitored simultaneously. When the sheet temperature was −72 °C, the absolute temperature difference |Δ*T*| values of the one-layer aerogel fiber mat, silk fabric mat, and cotton thread mat were 59 °C, 22 °C, and 46 °C, respectively. This demonstrates that CAF-3 has excellent insulation performance in cold environments.

### 2.5. Thermal Stability

**Figure 7:**
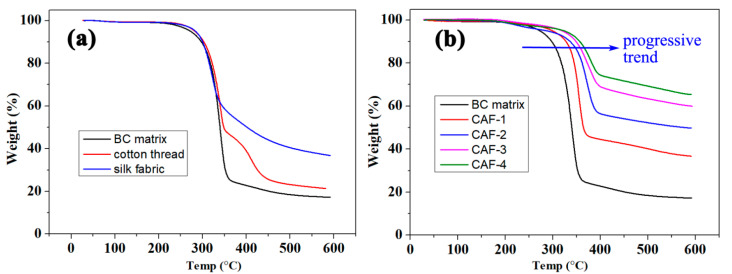
(**a**) Thermogravimetry analysis (TGA, 10 °C min^−1^ heating) curves of BC matrix, cotton thread, and silk fabric. (**b**) Thermogravimetry analysis (TGA, 10 °C min^−1^ heating) curves of BC matrix and CAFs. The blue arrow shows the temperature changes of each material at the same change in weight proportion (88%).

As shown in Figure 7a, the BC matrix demonstrates thermal stability similar to that of cotton thread and silk fabric, which are also composites of natural polymers. The thermogravimetric analysis curves showed that the temperature of decomposition of the cellulose (BC matrix) in composite aerogel fibers gradually shifted from about 280 °C to about 330 °C (Figure 7b) as the silica content increased. Moreover, when the mass gradually decreased to 88% of the initial mass, as shown by the blue arrow in Figure 7b, the temperature corresponding to the sample gradually rose from 300 °C to about 370 °C as the silica content increased. Hence, silica improved the thermal stability of the polymer matrix. This allows the CAFs to be used at higher temperatures than the traditional polymer fiber, owing to the stabilizing effect of silica on BC matrix.

## 3. Conclusions

In summary, a novel and simple method for preparing aerogel fibers with excellent mechanical properties and thermal insulation has been demonstrated here. The silica precursor TEOS and BC matrix were used to obtain the CAFs through in situ sol–gel reaction. The mechanical properties of CAFs were significantly improved by increasing the content of BC nanofibers per unit volume via a secondary shaping process. Efficient silica gel skeleton formed in the BC matrix endowed the CAFs with excellent thermal insulation performance and large specific surface area (606.9 m^2^/g). Moreover, the stabilizing effect of silica on the BC matrix makes the CAFs usable at relatively high temperatures (up to about 330 °C). In addition, the outstanding hydrophobicity of CAFs enables them to resist erosion by water vapor and have good weather resistance. Consequently, these aerogel fibers are promising materials for wearable thermal insulation. This work introduced the nanoscale interpenetrating network structure into aerogel fibers and provided a new way for the preparation and toughening of aerogel fibers, especially the inorganic oxide-based aerogel fibers.

## 4. Materials and Methods

### 4.1. Materials

*Nata-de-coco* slices were purchased from Wenchang Baocheng Industry and Trade Co., Ltd. (Hainan, China). Tetraethoxysilane (TEOS), *n*-hexane, triethylamine (TEA), trimethylchlorosilane (TMCS), and tert-butanol were obtained from Aladdin Reagent Co., Ltd. (Shanghai, China). Ethanol, hydrochloric acid (HCl), sodium hydroxide (NaOH, 4 wt %), and ammonium hydroxide (NH_3_·H_2_O) were purchased from Beijing Chemical Reagent Co. (Beijing, China), LTD. All the chemicals were of analytical grade and were used as received without any further purification.

### 4.2. Preparation of BC Fibers

**Figure 8:**
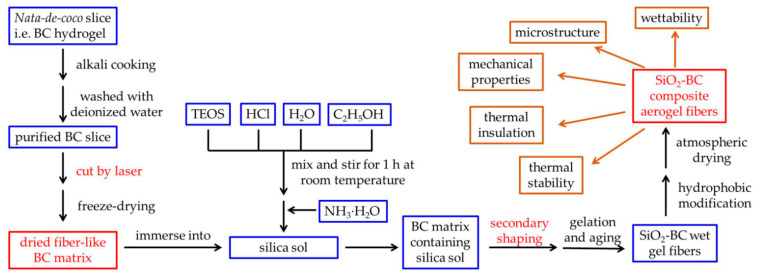
Flowchart illustrating the overall processes used in this work.

*Nata-de-coco* slices (i.e., bacterial cellulose hydrogel, BC hydrogel) of thicknesses of 3 mm were first soaked in deionized water for 4 h. The water was replaced several times to remove the added sucrose; after this process, the slices were boiled in NaOH for 6 h at 90 °C. Subsequently, clean BC hydrogel was obtained by rinsing it with deionized water until it became neutral. The *nata-de-coco* slice (Figure S1a) was placed on a glass plate, squeezed to remove about 80% of the water, and then cut using a laser (15 W power, 80W6040, Liaocheng Julong Laser Equipment Co., Ltd., Liaocheng, China) to obtain fibers of uniform width (2 mm) and length (about 500 mm) (Figure 1a and Figure S1b). Finally, the dried fiber-like BC matrix (Figure 1b and Figure S1c) was obtained by freeze drying for 24 h after the solvent replacement of the mixed liquid of water and tert-butanol (V_water_:V_tert-butanol_ = 3:2). This step was shown on the left side of Figure 8.

### 4.3. Preparation of Silica Sols

The preparation of silica sols with different concentrations of precursor TEOS was carried out by following the previously reported method [40]. The dosages of deionized water, HCl (*w*/*w* 1%), and ethanol in the process of sol preparation were 2, 0.2, and 9.2 mL, respectively. Different amounts (Table 2) of TEOS, deionized water, ethanol, and HCl were mixed in a beaker and stirred for 1 h at room temperature. Dilute ammonia (0.1 mol L^−1^, 1 mL) was added to this system to form silica alcosols as shown in the middle of Figure 8.

### 4.4. Preparation of Silica–BC Composite Wet Gel Fibers

The dried fiber-like BC matrix was immersed in silica sol and then stirred (Figure 1c and Figure S1d). The silica sol with the matrix was placed in an ice-water bath to inhibit the gelation process, ensuring that the silica sol could fully diffuse into the BC matrix. After sufficient diffusion for 2 h, the BC matrix containing silica sol was removed and passed through a tapered mold (the front end of 1000 μL pipette gun head with an inner diameter of 0.8 mm was cut as mold, produced by Nantong Hairui Experimental Equipment Co., Ltd., Nantong, China) immediately to make the fiber-like BC matrix finer and more uniform (Figure 1d and Figure S1e); this was the secondary shaping process. Then, the fiber-like BC matrix was placed in a sealed container filled with ethanol vapor for approximately 25 min to obtain silica–BC composite wet gel fibers (Figure 1e). The ethanol vapor was used to ensure that the composite wet gel did not shrink.

### 4.5. Hydrophobic Modification and Atmospheric Drying of CAFs

The wet gel fibers were soaked in ethanol and heated at 70 °C for 1 h to age the silica gel skeleton. Then, ethanol was replaced with *n*-hexane for 3 h for solvent replacement. *N*-hexane (50 mL), TEA (4 mL), and TMCS (3 mL) were added to the glass reactor, and the wet gel fibers were immersed in the liquid mixture. The glass reactor was heated in an oil bath and refluxed for 2 h. Then, the wet gel fibers were dipped into a 100 mL beaker containing 50 mL ethanol, which was replaced every 30 min; this process was repeated twice. Subsequently, ethanol was replaced with *n*-hexane, and the same operation as above was repeated. Subsequently, the wet gel fibers after hydrophobic modification were removed and heated in an oven at 80 °C for 20 min. Finally, the hydrophobic CAFs were obtained (Figure 1f and Figure S1f).

### 4.6. Characterization

As shown in the upper right corner of Figure 8, the morphology of the CAFs was determined, and the specific surface area, porosity, pore-size distribution, mechanical properties, wettability, density, and content of silica in the CAFs were examined. Thermal insulation performance and thermal stability were also evaluated. Detailed characterization methods are provided in the Supporting Information.

## Figures and Tables

**Table 1:** Physical properties of CAFs.

Samples	SiO_2_ in Aerogels [% *w*/*w*]	Bulk Density [g cm^−3^]	S_BET_ [m^2^ g^−1^]	Pore Size [nm]	Porosity ^a^ [%]
CAF-1	27	0.110	367.9	14.2	93.6
CAF-2	40	0.121	387.6	13.7	93.3
CAF-3	49	0.143	541.1	15.5	92.2
CAF-4	55	0.164	606.9	15.1	91.2

^a^ The porosity includes the voids caused by crystal growth among gel skeletons during gel freezing.

**Table 2:** CAFs formed by varying concentration of precursor TEOS.

Sample	CAF-1	CAF-2	CAF-3	CAF-4
TEOS (mL)	0.85	1.7	2.55	3.4

## Supplementary Materials

The following are available online at https://www.mdpi.com/article/10.3390/gels7030145/s1, Video S1: video of the wettability of the silica–bacterial cellulose composite aerogel fibers (CAFs), Figure S1: Photos of the preparation process of CAFs; Figure S2: SEM images of BC without secondary shaping; Figure S3: Temperature–time curves of CAFs at high temperature; Characterization and Reference.
