# Supplementary material for: Robust Silica-Bacterial Cellulose Composite Aerogel Fibers for Thermal Insulation Textile
Source: Gels. 2021 Sep 17;7(3):145. doi: 10.3390/gels7030145 (PMC8482140; doi:10.3390/gels7030145)
Supplement: Supplementary file 1 [file gels-07-00145-s001.zip › gels-1360621-for conversion-supplementary.pdf]

gels

MDPI

Article

Robust Silica-Bacterial Cellulose Composite Aerogel Fibers for Thermal Insulation Textile

Huazheng Sai [1,2,3,*], Meijuan Wang [1,2,3], Changqing Miao [1,2,3], Qiqi Song [1,2,3], Yutong Wang [1,2,3], Rui Fu [1,2,3,*], Yaxiong Wang [1,2,3], Litong Ma [1,2,3] and Yan Hao [1,2,3]

Supplementary Information (SI)

This Supporting Information contains:

1. Supplementary video

2. Characterization

3. The photos of the preparation process of CAFs

4. SEM images of BC without secondary shaping

5. Temperature-time curves of CAFs at high temperature

6. Reference

1. Supplementary Video

Video S1

Video of the wettability of the silica-bacterial cellulose composite aerogel fibers (CAFs).

2. Characterization

Characterization on Micromorphology: The micromorphology of the products were observed using scanning electron microscopy (SEM, SIGMA500, ZEISS, Germany) at an acceleration voltage of 3kV after sputtering with gold for its porous structure and insulation.

Characterization on Mechanical Properties: The mechanical properties of the fibers were tested by an electronic universal testing machine (HD-B609B-S, Guangdong, China), in the tensile mode. The strain rate was 2 mm/min. All the test samples were 2 cm. Each set of tensile strength test results are collected from at least five samples to obtain reliable values.

Density measurement: Take the CAFs sample of 5 cm length, combined with the diameter given by SEM images to calculate the total volume of the sample (Vc). Its mass (mc) was measured by a balance with a precision of 0.0001 g. The density of CAFs is calculated according to the ratio of mass to volume (mc/Vc).

Nitrogen physisorption measurement: The specific surface aera of the fibers were measured by the Brunauer-Emmett-Teller (BET) method in the condition of the Nitrogen adsorption at pressures 0.01 $<$ p/p0 $<$ 1.0. The Barrett-Joyner-Halenda (BJH) Analyses were conducted from desorption isotherm when the pore-size distribution was investigated. Content of silica in the CAFs: The dried BC matrix of 5 cm in length was intercepted, its mass was weighed (m0), Mass fraction (ω_s) of silica could be calculated by the equation:

$$\text{Mass fraction } (\omega_s) = \frac{m_C - m_0}{m_C} \quad (1)$$

Porosity measurement: The porosity of CAFs was calculated according to equation 2, where ϱ, ϱs and ϱc are the bulk density of CAFs, the skeleton densities of pure silica aerogels and biopolymer (i.e. BC) matrix; ωs and ωc were the mass fraction of silica and BC in CAFs, respectively. Herein, based on literature data, the ϱs and ϱc were designed as 2.1 g cm−3 and 1.59 g cm−3 [1].

$$\text{Porosity } (\%) = \left(1 - \frac{\rho}{\omega_s \rho_s + \omega_c \rho_c}\right) \times 100 \quad (2)$$

Wettability test: The wettability of the sample was tested by video optical contact angle measuring instrument (OCA25L, DataPhysics Instruments GmbH, Filderstadt, Germany). Firstly, about 3 μL of deionized water was extruded from the syringe and hung at the tip of the needle. The wettability of the sample was observed during the process as it contacts the water droplet and then leaves the water droplet.

Thermal insulation measurement: The thermographs were obtained by a thermal infrared camera (FLIR T620, Teledyne FLIR, Woburn, MA, USA). The camera was operated at a distance of about 30 cm. The CAFs sample were put on the heating plate, one thermo couple was connected to the sample and the other was on the hotplate next to the sample. Gradually heating up, the date acquisition device (34972A, Agilent, Santa Clara, CA, USA) was used to record the temperature of two thermo couples simultaneously.

Thermal stability: Thermogravimetric analysis of the sampales was carried out by a NETZSCH STA 449C (Erich NETZSCH GmbH & Co. Holding KG, Gebrüder, Germany). The samples were placed in a platinum pan and heated from 25 to 600 oC at a rate of 10 K min-1 under a nitrogen atmosphere.

3. The photos of the preparation process of CAFs

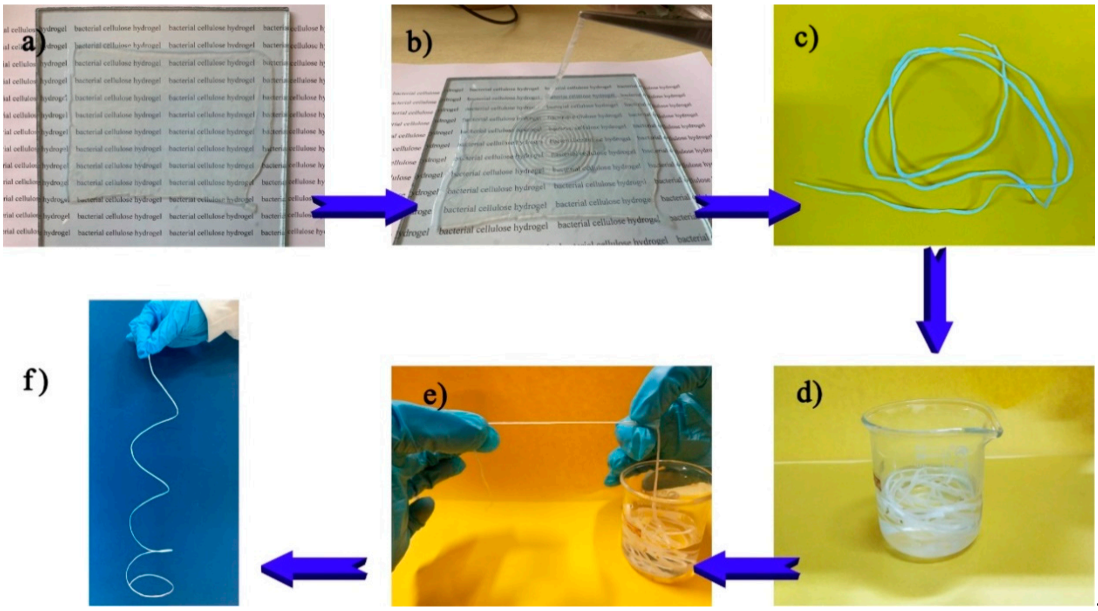

Figure S1. The photos of the preparation process of CAFs: **(a)** BC hydrogel slice, **(b)** fiber-like BC hydrogel, **(c)** dried fiber-like BC matrix, **(d)** the immersion of BC in silica sols, **(e)** the secondary shaping of the BC matrix containing silica sols and **(f)** the sample of CAF.

4. SEM images of BC without secondary shaping

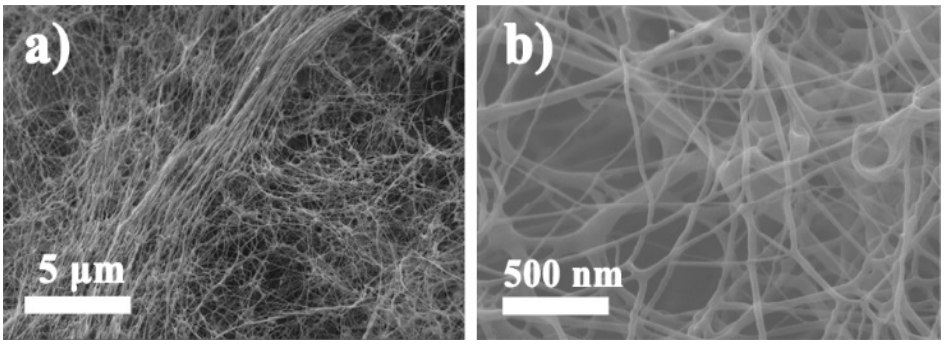

Figure S2. SEM images of BC matrix without secondary shaping.

5. Temperature-time curves of CAFs at high temperature

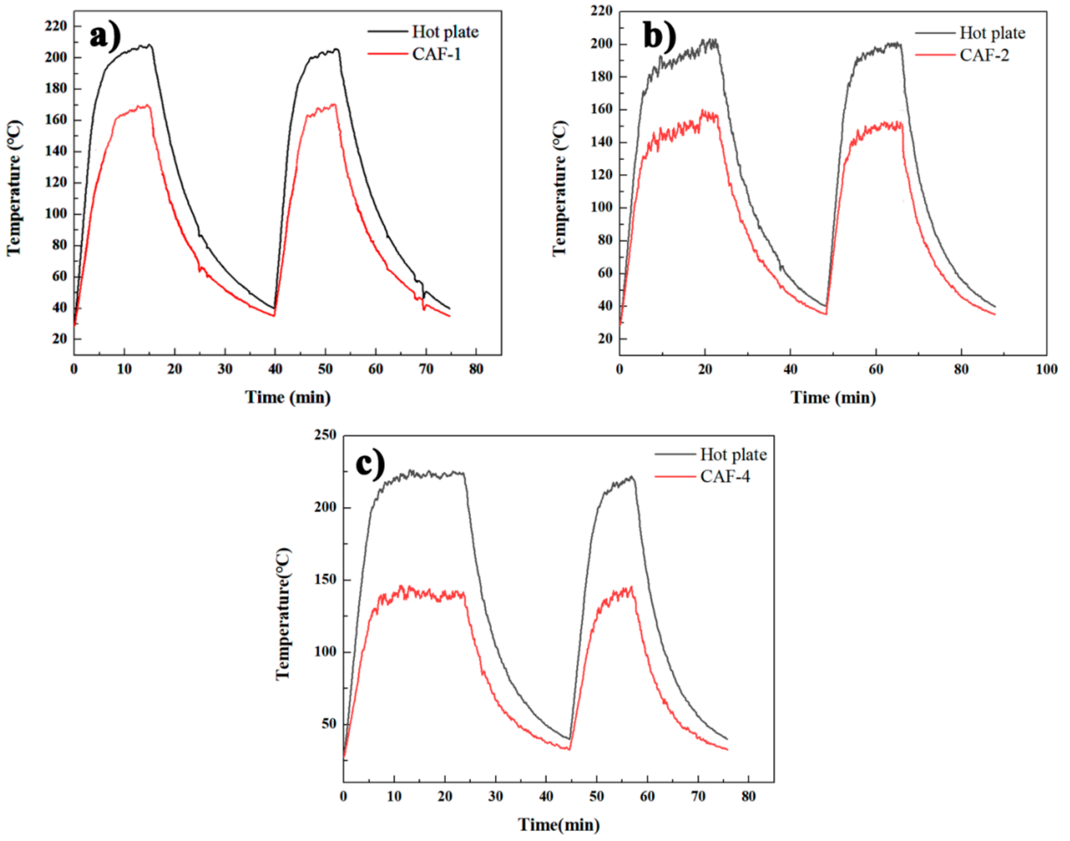

Figure S3. Temperature-time curves of CAF-1 **(a)**, CAF-2 **(b)** and CAF-4 **(c)** at high temperature (210 °C).

6. Reference

[1] Heath, L.; Thielemans, W. Cellulose nanowhisker aerogels. *Green Chem*. **2010**, 12, 1448−1453.
